# Reduction of Adolescent Idiopathic Scoliosis Utilizing the Labyrinthine Righting Reflex: A Case Report

**DOI:** 10.7759/cureus.101343

**Published:** 2026-01-12

**Authors:** Justin M Dick, John Whelan

**Affiliations:** 1 Physical Medicine and Rehabilitation, Clear Life Scoliosis and Chiropractic Center, Charlotte, USA

**Keywords:** idiopathic scoliosis, scoliosis management, scoliosis progression, sensory motor reflex, vestibulo-spinal reflex

## Abstract

Adolescent idiopathic scoliosis (AIS) remains a significant clinical challenge due to limited nonsurgical interventions and inconsistent treatment protocols. Current standard practices, including observation, rigid bracing, and spinal fusion surgery, often overlook patients with mild to moderate curvature, contributing to progression and decreased quality of life. This retrospective single case report evaluates a nonsurgical treatment integrating a cantilever traction system, weekly spinal mobilization, and reflexive engagement of the labyrinthine righting reflex (LRR) in an adolescent female patient. Following three months of intervention, the patient's mild Cobb angle reduced from 10.9° to 3.8°, accompanied by clinical improvements in posture, neuromuscular balance, and proprioceptive awareness. These observations suggest the potential of integrated conservative interventions addressing existing gaps in scoliosis care, particularly for mild AIS patients.

## Introduction

Adolescent idiopathic scoliosis (AIS) is defined as a lateral spinal curvature exceeding 10° with vertebral rotation, affecting approximately 2-3% of adolescents, predominantly females [1]. The female-to-male ratio in AIS ranges from 1.5:1 to 3:1 and increases significantly with age [2]. In the Netherlands, the annual societal cost per AIS patient was estimated at $13,332 USD, with healthcare expenditures and productivity losses accounting for the largest share of these costs [3].

AIS management traditionally stratifies interventions into observation for mild curves, orthotic bracing for moderate curves, and spinal fusion surgery for severe progressive curves [4]. Non-surgical management of AIS may involve physiotherapeutic scoliosis-specific exercises (PSSEs) for curves less than 25°, while combined PSSE and bracing is recommended for curves measuring approximately 25° to 60° [5,6,7]. The limitations of these approaches, including the restrictive nature of bracing and the invasiveness of surgery, highlight the need for alternative non-surgical methods, especially for individuals with mild-to-moderate scoliosis. Observation of curves under 25 degrees is recommended for AIS that are skeletally mature or curves that are not progressing [8]. 

Notably, underserved are patients with mild-to-moderate scoliosis who frequently progress unnoticed or inadequately managed until the severity mandates invasive intervention. The lack of widely adopted nonsurgical methods addressing early-stage spinal deformity represents a critical gap in current AIS management [9].

PSSE has produced positive results in mild to moderate scoliosis. PSSE are individualized, non-surgical rehabilitation programs designed to address the three-dimensional nature of scoliosis through curve-specific postural correction. PSSE incorporates active three-dimensional auto-correction, rotational breathing techniques, neuromuscular stabilization, and sensorimotor training to improve spinal alignment, trunk symmetry, and postural control. PSSEs are most commonly recommended for patients with mild AIS or as an adjunct to bracing in moderate curves [10].

A growing body of evidence utilizing the CLEAR Scoliosis Institute's protocols offers a nonsurgical scoliosis approach integrating chiropractic adjustments, neuromuscular reeducation, vibration therapy, and spinal traction [11]. The CLEAR Scoliosis Institute utilizes a multimodal approach incorporating body weighting, inducing the labyrinthine righting reflex (LRR). 

The LRR is a vestibular-mediated postural reflex arising from the otolith organs of the inner ear that contributes to the maintenance of head position relative to gravity. Alterations in head orientation are sensed by the vestibular system and processed through brainstem and cerebellar pathways, eliciting reflexive activation of cervical and truncal musculature to support upright posture and visual stabilization. Prior experimental and clinical investigations have established the importance of vestibular input in postural control and regulation of axial muscle tone under gravitational loading. It has been proposed that controlled activation of vestibular righting responses may influence neuromuscular coordination and postural organization, with potential secondary effects on spinal alignment mediated by changes in muscle activation patterns rather than direct structural modification [12,13,14]. Moreover, incorporating LRR may represent a critical innovation in conservative care, facilitating sustained correction through neurophysiological retraining [15].

This report details a retrospective single-patient case exploring a conservative approach incorporating LRR activation in mild AIS.

## Case presentation

On May 8, 2025, a 17-year-old female presented directly to a chiropractic clinic with complaints of postural asymmetry and right-sided low back pain. She had not undergone prior medical evaluation for this condition and reported no symptomatic improvement following previous infrared sauna therapy. The subject had a history of chiropractic care, with the most recent treatment occurring approximately three months before presentation. No diagnostic imaging had been obtained prior to evaluation at our clinic. The subject denied abdominal pain or tenderness, fever, bowel or bladder dysfunction, and radiating/numb pain to the upper or lower extremities.

The subject presented with no family history of scoliosis. The subject had daily right low back pain that increased as the day progressed. The subject reported increased pain with sitting for over 30 minutes. Pain improved with rest and did not interfere with activities of daily living. The subject denied any other chronic medical conditions. The subject denied any initiating factors of this pain.

The subject's blood pressure measured 117/82 mmHg with a heart rate of 62 beats per minute. Visual inspection of the subject revealed left head tilt with right rotation and translation, a right low hip, and a right low shoulder. The subject presented with a left translation of the thoracic spine. Palpation revealed areas of muscular spasm, joint hypomobility, and end-range tenderness consistent with segmental dysfunction at left T1, left T2, right T3, right T4, right T5, right T6, right T7, right T8, right T9, right T10, right T11, right T12, right L1, right L2, right L3, right L4, right L5, left sacrum, and left pelvis. As scoliosis is a lateral curve accompanied by rotation of the vertebra, these rotation findings can be commonly found in scoliosis. Table 1 presents the physical and orthopedic examination results.

**Table 1:** Physical examination results after the 90-day treatment plan The Functional Rating Index (FRI) combines the content of the Oswestry Low Back Disability Questionnaire and the Neck Disability Index in a format that reduces administrative burden. FRI has recently been tested, and the results have been published in Spine (Feise, 2001). Based on the initial research, the FRI demonstrates excellent reliability, validity, and responsiveness, and significantly reduces administrative burden [16]. Permission was obtained from the original publisher to reproduce this table [17].

Assessment parameter	Pre-treatment	90-day follow-up
Forward head posture	1.0 inch	1.0 inch
Angle of trunk rotation – dorsal (flexion)	0°	0°
Angle of trunk rotation – dorsal–lumbar (flexion)	5° right	2° right
Angle of trunk rotation – lumbar (flexion)	5° right	2° right
Angle of trunk rotation – dorsal (prone)	0°	0°
Angle of trunk rotation – dorsal–lumbar (prone)	5° right	2° right
Angle of trunk rotation – lumbar (prone)	10° right	3° right
Thoracic lordotization level	T1–T12	T1–T12
Cervical flexion test (chin-to-chest distance)	1.0 inch	0.0 inches
Modified Scoliosis Cox Test – left	Positive at 75°	Positive at 90°
Modified Scoliosis Cox Test – right	Positive at 75°	Positive at 90°
Balance testing	Within normal limits bilaterally	Within normal limits bilaterally
Stork Test – left (seconds to failure)	8 seconds	13 seconds
Stork Test – right (seconds to failure)	12 seconds	20 seconds
Kemp’s Test	Negative	Negative
Straight Leg Raise Test	Negative	Negative
Functional Rating Index (FRI)	9/40	3/40

Initial radiographs

Standing radiographs were obtained in compliance with U.S. Medicare guidelines, with the subject positioned upright, weight-bearing, and in a neutral posture. Scoliosis radiographs were obtained and digitized using the PostureRay® EMR system (Trinity, FL, USA), a validated imaging analysis platform. Coronal alignment was measured using the Risser-Ferguson method. Initial radiographs revealed a right lumbar Cobb angle measurement of 10.9°, classifying the subject as mild AIS.

When interpreting the magnitude of radiographic change, inherent measurement variability associated with Cobb angle assessment must be considered. Prior investigations have demonstrated high intra- and inter-examiner reliability for standardized radiographic line-drawing methods, with reported measurement error generally ranging between ±3° [18,19]. 

Accordingly, changes exceeding this threshold are typically regarded as clinically meaningful, whereas smaller variations may reflect normal measurement variability rather than true structural change. A change in Cobb angle is deemed significant if measured over 5 degrees [5]. 

Treatment rationale

Due to the subject's skeletal maturity, Risser 5, and mild spinal curvature, conservative nonsurgical intervention was prioritized.

Methods

Institutional Review Board (IRB) approval was not required for this study due to its retrospective nature and use of de-identified data, in accordance with the U.S. Common Rule (45 CFR 46.104). Standard written informed consent was obtained from the subject. No identifiable personal or protected health information is included.

The intervention protocol consisted of weekly in-office chiropractic spinal adjustments, daily home-based cantilever traction using a Skolios (Charlotte, NC, USA) cantilever system with proprioceptive neuromuscular reeducation emphasizing LRR activation. 

The cantilever system was applied to deliver translational and rotational forces at the radiographically identified apex, verified using anteroposterior lumbopelvic radiographs. The device provides a pivot point designed to intensify reflexive postural responses through engagement of the labyrinthine system. A measured force of 3 pounds at the distal end of the effort arm.

Manual therapy adjustments were administered to address joint dysfunction, muscular imbalance, and neural restriction, with a total of 11 manual therapies delivered over a three-month time period.

**Figure 1:**
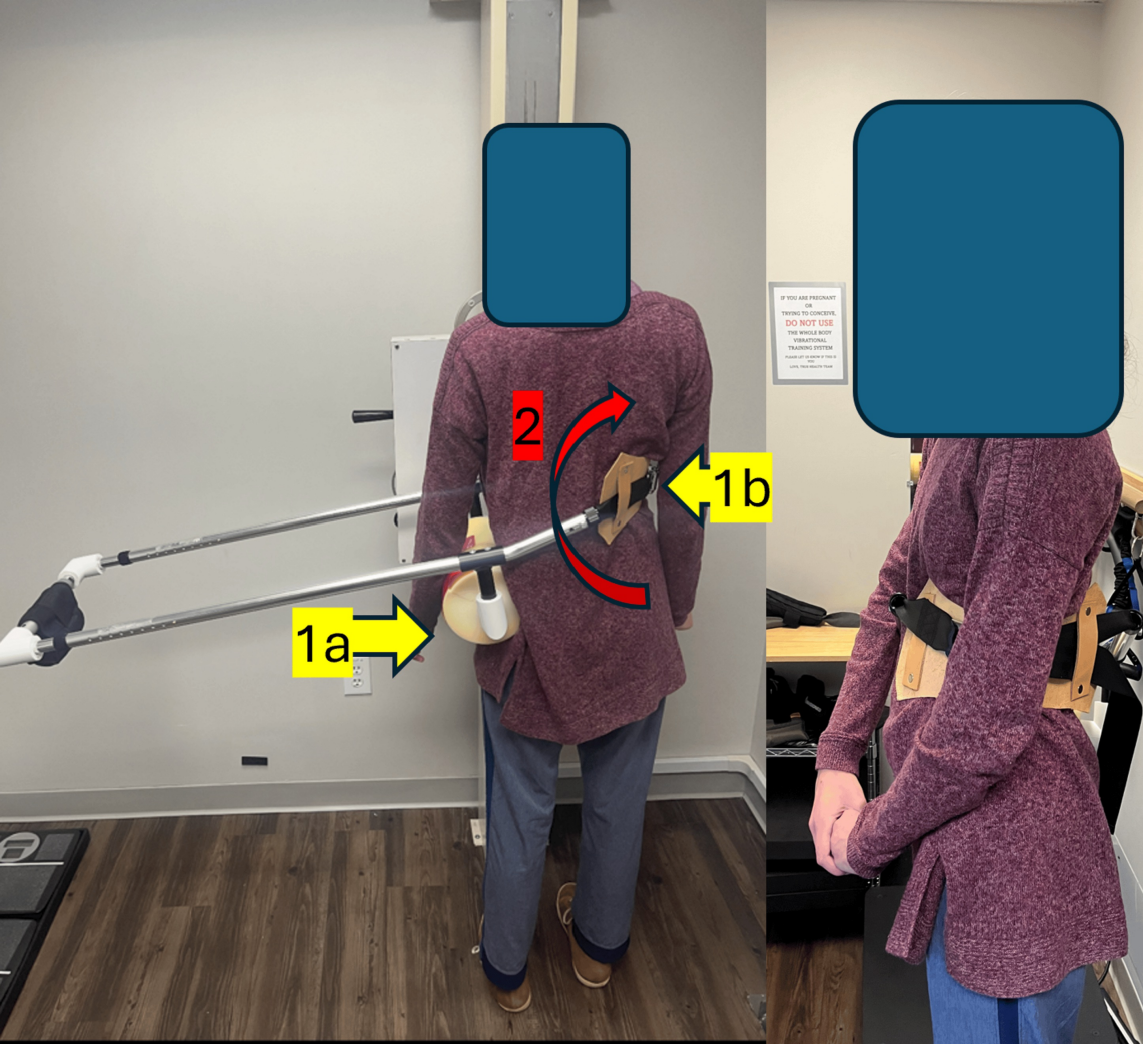
Spinal weighting (proprioceptive neuromuscular re-education, model used for demonstration) Spinal weighting was performed using a proprioceptive neuromuscular re-education, cantilever system. The subject performed balance and proprioceptive exercises on unstable surfaces, including a foam pad and a vibration platform, to promote postural correction, coordination, and kinesthetic awareness. This intervention targets balance impairments related to neuromuscular or musculoskeletal dysfunction. Treatment was performed for approximately ten minutes per session, two sessions daily for home use. 1: Passive forces, downward force on the 1a side, and push on the 1b side. 2: Active reaction using the long-latency reflex (LLR) reaction, flexion away from the weight above pivot point.

Cantilever traction sessions were prescribed twice daily for 10 minutes, performed on a balance pad to further challenge postural stability and enhance reflexive engagement. In-office proprioceptive training incorporated uneven spinal weighting on an unstable and vibrating surface to target coordination, kinesthetic awareness, and neuromuscular control, as shown in Figure 1.

Results

Radiographic Outcomes

**Figure 2:**
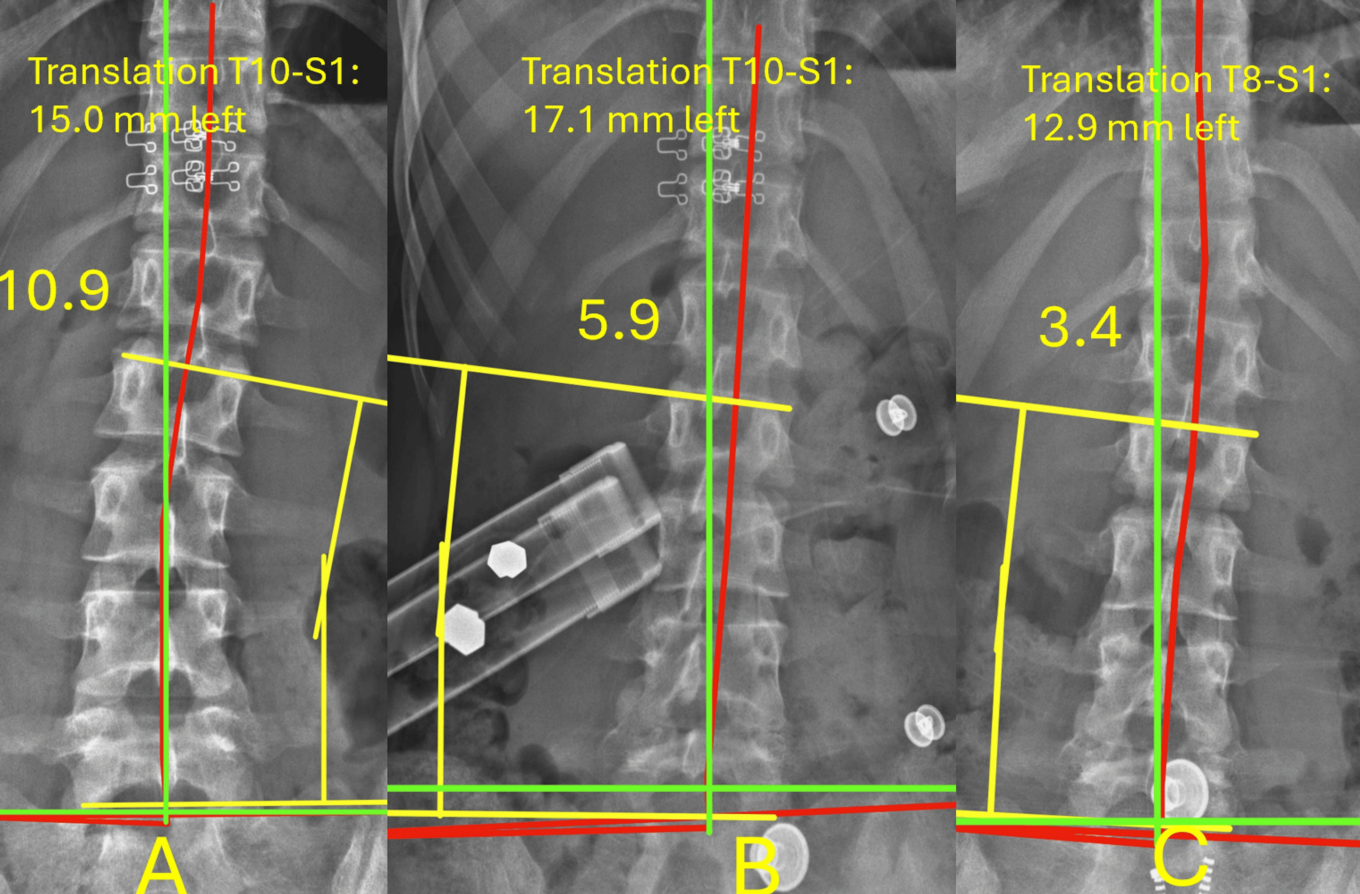
Anterior-posterior scoliosis radiographs Anterior-posterior spine radiographs. Panel A is upon initial presentation. Panel B is a stress radiograph with cantilever. Panel C is the 90 day follow up radiograph. The red lines represent the Risser–Ferguson analysis of the subject's spine, demonstrating improvement in Cobb angle measurements and overall coronal balance.

Radiographs at baseline demonstrated an initial Cobb angle of 10.9°. After three months, follow-up radiographs revealed a significant reduction to 3.4°, equating to a 31% improvement. Thoracic translation was reduced from 15.0 mm to 12.9 mm, equating to 15% improvement, as shown in Figure 2.

Clinical Outcomes

Clinically, the subject exhibited significant postural improvement, symmetrical muscular activation patterns, enhanced proprioceptive accuracy, and overall subjective improvements in pain, daily functioning, and physical activities. The subject's FRI initially presented as a 9/40 and decreased to a 3/40, a 15% improvement. 

## Discussion

Currently, nonsurgical AIS interventions are inadequately emphasized, often limited to rigid immobilization bracing. These approaches ignore essential proprioceptive and neuromuscular training elements, necessary for sustainable correction, leading many patients toward progression and surgical intervention unnecessarily [5,7]. Ineffective treatments lead to an undue burden on those suffering from scoliosis.

Ingles et al. examined the critical role of somatosensory input in postural control, with a focus on automatic and righting responses. It highlights how proprioceptive impairments can disrupt righting reflexes, which are essential for maintaining balance and upright posture. The findings are particularly relevant to the understanding and treatment of balance disorders, neurological impairments, and rehabilitation strategies [20]. The role of somatosensory input in initiating and modulating automatic postural responses in humans [21]. Studies have also shown that proprioceptive deficits are consistently present in individuals with AIS [22].

The CLEAR Scoliosis Institute utilizes a specific treatment tool with patients with AIS, which involves utilizing a cantilever to apply force to a specific X-ray-guided area [17,23]. We hypothesize that the cantilever system activates the LRR. The benefit of the cantilever use is that it can apply translational and rotational forces while simultaneously providing a pivot point, increasing the intensity of the LRR.

This novel approach represents a significant departure from conventional allopathic interventions, particularly for mild to moderate scoliosis, by leveraging the body's intrinsic physiological mechanisms for postural realignment. Specifically, the cantilever device aims to stimulate the righting reflex, an involuntary response that helps maintain upright posture and balance, to guide the spine towards a more anatomically correct alignment [24]. This biofeedback-driven mechanism facilitates continuous muscular engagement and proprioceptive awareness, which are crucial for sustained postural correction beyond the active treatment session [25].

This case highlights how an integrative conservative approach using spinal adjustments and cantilever traction successfully addresses mild AIS by engaging neurophysiological mechanisms, primarily the labyrinthine righting reflex. Unlike standard bracing methods, this combined intervention promotes active muscular strengthening, proprioceptive training, and sustained neuromuscular adaptation, crucial elements often overlooked in nonsurgical scoliosis management.

This case is limited by its single-patient design and short three-month duration, which restricts the generalizability of the findings. As such, the outcomes observed should not be assumed to apply broadly to all cases of adolescent idiopathic scoliosis. Based on these findings, continued care is likely warranted. 

## Conclusions

This case report presents a unique application of the LRR incorporated may give an improvement in a scoliotic deformity, reduce thoracic translation, and alleviate lumbar pain in a 17-year-old female.

Integrating weekly mobilization and a cantilever spinal traction may offer an alternative for managing mild-to-moderate AIS cases. This case underscores the clinical value of radiographic assessment for precise diagnosis and biomechanical evaluation in adolescent scoliosis. Although limited to a single subject, it highlights the need for continued research into non-surgical scoliosis interventions, particularly those targeting individualized biomechanical correction in a population where high-quality evidence remains limited. Prospective, longitudinal, clinical trials are needed to further investigate and validate the clinical utility and reproducibility of this integrative protocol.
